# Secondary Infections in Critically Ill Patients with COVID-19: A Retrospective Study

**DOI:** 10.3390/antibiotics11111598

**Published:** 2022-11-11

**Authors:** Luca Caiazzo, Chiara Temperoni, Benedetta Canovari, Oriana Simonetti, Roberto Montalti, Francesco Barchiesi

**Affiliations:** 1 Malattie Infettive, Azienda Ospedaliera Ospedali Riuniti Marche Nord, 61121 Pesaro, Italy; 2 Clinica Dermatologica, Dipartimento di Scienze Cliniche e Molecolari, Università Politecnica delle Marche, 60126 Ancona, Italy; 3 Unità di Chirurgia Epato-Bilio-Pancreatica, Mininvasiva e Robotica, Dipartimento di Sanità Pubblica, Università Federico II, 80131 Napoli, Italy; 4 Dipartimento di Scienze Biomediche e Sanità Pubblica, Università Politecnica delle Marche, 60126 Ancona, Italy

**Keywords:** COVID-19, mechanical ventilation, hospital-acquired infections, resistance

## Abstract

Patients with severe COVID-19, especially those followed in the ICU, are at risk for developing bacterial and fungal superinfections. In this study, we aimed to describe the burden of hospital-acquired superinfections in a cohort of consecutive, severe COVID-19 patients hospitalized between February and May 2021 in the intensive care unit (ICU) department of San Salvatore Hospital in Pesaro, Italy. Among 89 patients considered, 68 (76.4%) acquired a secondary infection during their ICU stay. A total of 46 cases of ventilator-associated pneumonia (VAP), 31 bloodstream infections (BSIs) and 15 catheter-associated urinary tract infections (CAUTIs) were diagnosed. Overall mortality during ICU stay was 48%. A multivariate analysis showed that factors independently associated with mortality were male gender (OR: 4.875, CI: 1.227–19.366, *p* = 0.024), higher BMI (OR: 4.938, CI:1.356–17.980, *p* = 0.015) and the presence of VAP (OR: 6.518, CI: 2.178–19.510, *p* = 0.001). Gram-negative bacteria accounted for most of the isolates (68.8%), followed by Gram-positive bacteria (25.8%) and fungi (5.3%). Over half of the infections (58%) were caused by MDR opportunistic pathogens. Factors that were independently associated with an increased risk of infections caused by an MDR pathogen were higher BMI (OR: 4.378, CI: 1.467–13.064, *p* = 0.0008) and a higher Charlson Comorbidity Index (OR: 3.451, 95% CI: 1.113–10.700, *p* = 0.032). Secondary infections represent a common and life-threatening complication in critically ill patients with COVID-19. Efforts to minimize the likelihood of acquiring such infections, often caused by difficult-to-treat MDR organisms—especially in some subgroups of patients with specific risk factors—must be pursued.

## 1. Introduction

Secondary infections have been commonly described in severe influenza, and they are responsible for increasing morbidity and mortality during pandemic and seasonal influenza episodes [1,2]. Patients with severe COVID-19, especially those followed in the intensive care unit (ICU), are at risk for developing bacterial and fungal superinfections.

Prolonged hospitalization either in general wards or in ICUs; the increased need of invasive procedures such as central venous catheter and mechanical ventilation (MV); and the use of broad-spectrum antibiotics and corticosteroids, play a key role and representing relevant risk factors for developing secondary infections often caused by multi-drug-resistant organisms (MDR) [3]. However, despite much being said on the risk factors, data about infections complicating COVID-19 are poor.

Therefore, in this study, we aimed to describe the burden of infections and identify the predictors of hospital-acquired superinfections in a cohort of consecutive, severe COVID-19 patients hospitalized during a four-months period in the ICU department of San Salvatore Hospital in Pesaro, Italy.

## 2. Results

A total of 89 patients were considered. The mean age of the patients was 68 years and 66 (74.2%) were male (Table 1). Most of the patients (78.7%) presented at least one comorbidity, with hypertension (52.8%), diabetes (19.1%) and chronic cardiac disease (18%) being the most common. Median body mass index (BMI) was 27. Over half of the population considered (57.3%) received antimicrobials prior to hospital admission. All patients were screened for MDR bacteria at admission, and none of them resulted positive for multi-drug-resistant enteric bacteria or methicillin-resistant *Staphylococcus aureus.*

Median hospital stay, ICU stay and duration of mechanical ventilation were, respectively: 20 (IQR 13–32), 19 (IQR 12–30) and 16 (IQR 10–28) days.

Sixty-eight out of 89 patients (76.4%) acquired a secondary infection during ICU stay, with 22 of them presenting with more than one infection. A total of 46 VAP cases, 31 bloodstream infections (BSIs) and 15 catheter-associated urinary tract infections (cUTIs) were diagnosed (Table 1). The incidence of hospital-acquired infection was 48.9 events per 1000 ICU days, with a VAP incidence of 26.2 events per 1000 ventilator days, The BSI incidence was 16.3 events per 1000 ICU days and cUTI incidence was 7.9 events per 1000 ICU days. Forty-three out of 89 patients (48.3%) died during the ICU stay. Death occurred at a median time of 15 days (IQR 10–23) from ICU admission. A multivariate analysis showed that factors independently associated with mortality were male gender (OR: 4.875, CI: 1.227–19.366, *p* = 0.024), higher BMI (OR: 4.938, CI:1.356–17.980, *p* = 0.015) and the presence of VAP (OR: 6.518, CI: 2.178–19.510, *p* = 0.001) (Table 1).

There were 21 out of 89 patients who did not experience any infection during the hospitalization. Comparing the two groups, we found that the factors independently associated with an increased risk of infections were higher BMI (OR: 4.284, CI: 1.409–13.032, *p* = 0.010) and higher CCI (OR: 3.540, 95% CI: 1.175–10.669, *p* = 0.025) (Table 2).

A total of 93 micro-organisms were identified as causative agents of secondary infections (Table 3). Gram-negative bacteria accounted for most of the isolates (68.8%), followed by Gram-positive bacteria (25.8%) and fungi (5.3%). There was a trend, although not statistically significant, towards a later onset of either VAP or BSI caused by Gram-negative than by Gram-positive bacteria: median time, 8 days (IQR 7–14) vs 5 days (IQR 4–8), respectively, for VAP (*p* = 0.09); 21 days (IQR 8–22) vs 7 days (IQR 2–9), respectively, for BSI (*p* = 0.056). The median onset of CAUTI was 9 days regardless of the type of bacteria.

Fifty-two out of 89 patients (58.4%) were infected with an MDR pathogen (Table 1 and Table 4) The remaining 37 patients were either infected with an MDS pathogen or did not experience any infection during hospitalization. Comparing the two groups, we found that the factors independently associated with an increased risk of infection caused by an MDR pathogen were higher BMI (OR: 4.378, CI: 1.467–13.064, *p* = 0.008) and a higher Charlson Comorbidity Index (CCI) (OR: 3.451, 95% CI: 1.113–10.700, *p* = 0.032).

**Figure 1:**
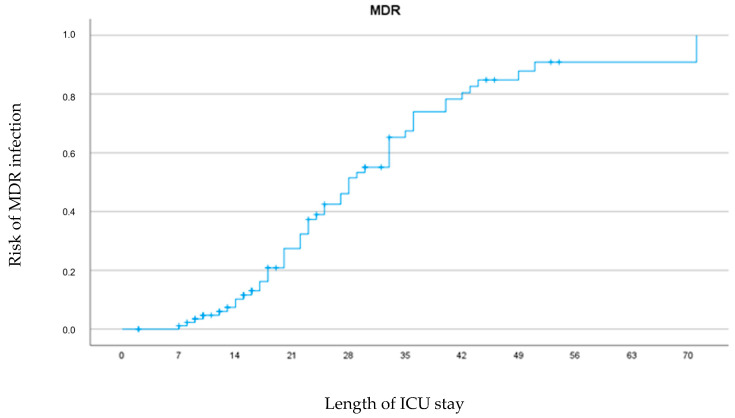
Relationship between length of stay and risk of MDR infection.

The longer the hospitalization time, the higher the risk of developing an infection due to an MDR pathogen (Figure 1).

## 3. Discussion

To date, most of the studies investigating secondary infections in COVID-19 patients have focused on the risk for VAP development [4,5,6,7]. Here, we describe the risk of any secondary infection in this group of patients under MV during the late second wave of SARS-CoV-2 in Italy. Even a year after the start of the pandemic, the demographic characteristics of our cohort are like those of early reports, with older age, male gender, hypertension and diabetes being the most common features. More than half of these patients experienced antibiotic over-use before ICU admission, even though the utility of antibiotics in SARS-CoV-2 therapy has not been demonstrated [4,7,8] 

All patients were undergoing antiviral therapy with remdesivir, steroids and anticoagulants.

Infections were reported in 76% of the overall population, with VAP, BSI and cUTI appearing in 52%, 35% and 17% of the patients, respectively. A similar distribution emerged in a large multi-centric retrospective Italian study, even though the overall frequency of infections was lower (66%) than in our sample [7]. Similarly, in large multi-centre studies, the frequency of secondary infections was somewhat lower (58%) than that found in our study [9,10]. This can be explained by the fact that 25% of our patients presented more than one infection during the ICU stay.

In general, Gram-negative bacteria represented the most common causative agents of infections seen in our patients. We found a distinct, rather constant temporal distribution between Gram-positive and -negative bacteria causing VAP and BSI, with the former yielding earlier infections. This characteristic has been already described in common influenza, where Gram-positive bacteria are responsible for early VAP, as they colonize the nasopharynx [1,11].

VAP was more frequently caused by *A. baumannii*, followed by *S. aureus* and *Klebsiella* spp. Differently from Ceccarelli et al. [12], we reported a few cases of Pseudomonas aeruginosa VAP. Contrarily to that reported in an Italian study [13], we found that MSSA causing VAP was more frequent than MRSA. In our series, the occurrence of VAP was independently associated with a higher risk of mortality, reaching 67%, which is higher than that reported in other studies [14,15]. Although the association between VAP and mortality has been investigated in several large clinical trials, it is difficult to identify the real cause of death in ICU patients, as a prolongated duration of MV and ICU stay could play a significant role in increasing the mortality risk beyond underlying medical conditions [16,17]. Male gender and a BMI > 26.7, besides VAP occurrence, are independently associated with a poor outcome.

The occurrence of BSI in our population did not increase the risk of mortality, contrary to the suggestion of another study [18]; the main causative agents of BSI were Gram-negative, such as *A. baumannii* and *Klebsiella* spp. Further, we found *Enterococcus* spp. in a small BSI percentage, unlike what was shown in an Italian study, which reported *Enterococcus* spp. to be the most common agent cultured from blood [8]. These differences can be explained by the variable hospital ecology, as well as by the selective pressure of specific antibiotics which can influence the microbial population [19].

The high frequency of *A. baumannii* infections in COVID-19 patients admitted to the ICU is a well-known problem [20,21]. One study evaluated the risk factors for MDR *A. baumannii* infections in ICU patients hospitalized for COVID-19 or other etiology and found that serum lactate levels mmol/L > 2, *A. baumannii* colonization, BSI and steroid therapy were observed more frequently in COVID-19 patients [21]. The high frequency of *A. baumannii* seen in our group of patients hypothesizes the occurrence of an outbreak which, along with a poor performance status, explains the high mortality rate observed in them. Due to a shortage of treatment options, infections due to *A. baumannii* are difficult to treat. A recent study analysed the impact of cefiderocol use on outcome in patients admitted to the ICU for severe COVID-19 and further diagnosed with carbapenem-resistant *A. baumannii* infection; they compared the use of cefiderocol versus colistin and found that this new drug was associated with a non-significant lower mortality risk [22]. Furthermore, it has been shown that cefiderocol has a greater activity than colistin against meropenem-resistant strains [23].

Here, we found that higher BMI and higher CCI were both associated with an increased risk of developing a secondary infection. Interestingly, the same two factors were significantly associated with an increased risk of developing MDR infections. Contrary to what might be inferred, pre-exposure to antibiotics did not influence the development of MDR infections.

It has been reported that the presence of comorbidities represents a risk factor of acquiring extended-spectrum beta-lactamase (ESBL)-producing bacteria [24]. In several studies, it has been shown that comorbidities play an important role in COVID-19 infected patients: two studies showed that CCI 1–2 in SARS-CoV-2 patients is related to poor outcomes and the risk for mortality increases by 16% for each increase in CCI [25,26]. However, the association between comorbidities presence and the risk of MDR infection is poorly investigated, although in SARS-CoV-2 negative patients VAP caused by MDR pathogens is mostly associated to prior exposure to more than two antibiotics and to the presence of comorbidities [27].

This study has several limitations. First, it is a retrospective monocentric study with a limited number of patients that may affect the analysis of the results. Second, over half of the patients had received an antibiotic treatment before ICU admission, which could have predisposed the presence of bacterial colonization. Third, our analysis could have been confounded by a high prevalence of *A. baumannii* infection, possibly due to an outbreak, even though we did not perform a molecular analysis, thereby influencing the high mortality rate found in our population. Furthermore, the data for the biochemical analysis performed at the entrance and during hospitalization, and for immune-modulating drugs, were not collected.

## 4. Materials and Methods

### 4.1. Study Design and Definitions

We conducted a single-centre, retrospective, observational study including all consecutive patients with COVID-19 admitted to the ICU department of San Salvatore Hospital in Pesaro, Italy, between 1 February and 31 May 2021, who underwent MV. All patients had a diagnosis of COVID-19 confirmed by real-time reverse transcription PCR performed on nasopharyngeal throat swab specimens.

Epidemiological and demographic data, medical history, comorbidities, and Charlson Comorbidity Index (CCI) at the time of hospitalization were collected. Data on antibiotic treatments received before hospital admission, length of hospital stay, and mortality were also obtained directly from medical records. The records of all patients with positive microbiologic results were reviewed by at least two infectious disease specialists to assess clinical significance. Infections occurring 48 h after hospital admission were categorized as bloodstream infection (BSI), ventilator-associated pneumonia (VAP) and catheter-associated urinary tract infection (cUTI), according to CDC definitions [28,29,30].

MDR was defined as non-susceptibility to at least one agent in three or more anti-microbial categories.

### 4.2. Study Objectives

The purposes of our study were to describe the clinical characteristics of COVID-19 patients under MV; describe the incidence of hospital-acquired infections (HAIs) such as VAP, BSI and UTI; and assess the risk of MDR pathogens-sustained infections and the risk factors for mortality.

### 4.3. Statistical Analysis

Normally distributed continuous data were reported as the mean ± SD and compared using the two-sided Student’s *t*-test. Non-normally distributed continuous data were reported as the median and IQR and compared using the Mann–Whitney test. Categorical variables were analysed with the χ^2^-test with Yates correction or Fisher’s exact test, whichever was most appropriate. A receiver operating curve (ROC) analysis was undertaken to identify a cut-off value for endpoint parameter. A logistic regression analysis with backward stepwise selection was constructed, employing those variables with a minimum number of 5 cases and a significance level of *p* < 0.20. IBM SPSS Statistics version 27 (SPSS, Chicago, IL, USA) was employed for the statistical analysis. Statistical significance was set at *p* < 0.05.

## 5. Conclusions

In conclusion, we found that secondary infections represent a common and life-threatening complication in critically ill patients with COVID-19. Efforts to minimize the likelihood of acquiring such infections, often caused by difficult-to-treat MDR organisms—especially in some subgroups of patients with specific risk factors—must be pursued.

## Figures and Tables

**Table 1:** Demographic, clinical characteristics and outcome of 89 COVID-19 patients considered in this study.

Characteristics	All Patients (*n* = 89)	Died (*n* = 43)	Survivor (*n* = 46)	*p*	OR (95% CI)	*p*
Gender (M)	66 (74.2%)	36 (83.7%)	30 (65.2%)	0.080	4.875 (1.227–19.366)	**0.024**
Mean age-ears	68.1 ± 9.3	70.1 ± 7.84	64.3 ± 9.69	0.003		
>70	34 (38.2%)	22 (51.2%)	12 (26.1%)	0.027	1.629 (0.586–4.532)	0.350
Median BMI (Body mass index)	27 (25.7–29.4)	27 (25.0–30.4)	27.7 (25.7–29.4)	0.511		
≥26	34 (38.2%)	20 (46.5%)	14 (30.4%)	0.180	4.938 (1.356–17.980)	**0.015**
Co-existing conditions						
Diabetes	17 (19.1%)	8 (18.6%)	9 (19.6%)	0.908		
Chronic/acute kidney disease	10 (11.2%)	8 (18.6%)	2 (4.3%)	0.045	3.098 (0.520–18.458)	0.214
Hypertension	47 (52.8%)	25 (58.1%)	22 (47.8%)	0.446		
Chronic obstructive pulmonary disease	6 (6.7%)	5 (11.6%)	1 (2.2%)	0.103	2.330 (0.233–23.291)	0.471
Heart disease	16 (18%)	6 (14%)	10 (21.7%)	0.497		
Malignancy	8 (9%)	4 (9.3%)	4 (8.7%)	>0.999		
Hematologic disease	3 (3.4%)	2 (4.7%)	1 (2.2%)	0.608		
Neurological disease and mental disorder	13 (14.6%)	4 (9.3%)	9 (19.6%)	0.285		
Charlson Comorbidity Index > 2	56 (63%)	31 (72.1%)	25 (54.3%)	0.130	1.064 (0.266–4.257)	0.931
Previous antibiotic treatment	51 (57.3%)	26 (60.5%)	25 (54.3%)	0.712		
Absence of microbial colonization	8 (9%)	6 (14%)	2 (4.3%)	0.149	5.452 (0.793–37.488)	0.085
Infection	68 (76.4%)	36 (83.7%)	32 (69.6%)	0.186	1.534 (0.295–7.994)	0.611
>1	22 (24.7%)	11 (25.6%)	11 (23.9%)	>0.999		
BSI	31 (34.8%)	15 (34.9%)	16 (34.8%)	>0.999		
VAP	46 (51.7%)	29 (67.4%)	17 (37%)	0.008	6.518 (2.178–19.510)	**0.001**
cUTI	15 (16.8%)	6 (14%)	9 (19.6%)	0.672		
MDR	52 (58.4%)	23 (53.5%)	29 (63%)	0.485		

Table 1 Data are expressed as mean ± SD, median (IQR) or *n* (%). *p*-values indicate differences between deceased and surviving patients. *p* < 0.05 was considered statistically significant. Statistically significant values are expressed in bold font. In round brackets are expressed percentages and IQR. BMI: body mass index; BSI: bloodstream infection; VAP: ventilator-associated pneumonia; cUTI: catheter-associated urinary tract infection.

**Table 2:** Risk factors for the onset of secondary infections.

Characteristics	Infection (68 pts)	Without Infection (21 pts)	*p*	OR (95% CI)	*p*
Gender (M)	51 (75%)	15 (71.4%)	0.967		
Age	67.6 ± 9.23	65.5 ± 9.45	0.375		
>70	28 (41.2%)	6 (28.6%)	0.434		
BMI	27.7 (25.7–31.0)	26.1 (25.0–27.2)	0.033		
>26.7	42 (61.8%)	7 (33.3%)	0.041	4.284 (1.409–13.032)	**0.010**
Previous antibiotic treatment	41 (60.3%)	10 (47.6%)	0.439		
Co-existing conditions					
Diabetes	15 (22.1%)	2 (9.5%)	0.341		
Chronic/acute kidney disease	10 (14.7%)	0 (0%)	0.109		
Hypertension	39 (57.4%)	8 (38.1%)	0.195	0.796 (0.241–2.632)	0.708
Chronic obstructive pulmonary disease	4 (5.9%)	2 (9.5%)	0.623		
Heart disease	14 (20.6%)	2 (9.5%)	0.340		
Malignancy	6(8.8%)	2 (9.5%)	>0.999		
Hematologic disease	1 (1.5%)	2 (9.5%)	0.137		
Neurological disease and mental disorder	12 (17.6%)	1 (4.8%)	0.286		
Charlson Comorbidity Index	3 (2–4)	2 (1.5–4.5)	0.112		
>2	48 (70.6%)	8 (38.1%)	0.015	3.540 (1.175–10.669)	**0.025**

Table 2 Data are expressed as mean ± SD, median (IQR) or *n* (%). *p*-values indicate differences between deceased and surviving patients. *p* < 0.05 was considered statistically significant. Statistically significant values are expressed in bold font. In round brackets are expressed percentages and IQR, as well as the subjects analysed.

**Table 3:** Opportunistic pathogens associated with secondary infections.

Micro-Organisms	Total (*n* = 92)	Bloodstream Infection (*n* = 31)	VAP (*n* = 46)	cUTI (*n* = 15)
Gram-positive cocci				
*Enterococcus faecalis*	8 (8.6%)	3 (9.7%)		5 (33%)
*Enterococcus faecium*	2 (2.1%)	2 (6.4%)		
*MSSA*	8 (8.6%)		8 (17.4%)	
*MRSA*	4 (3.2%)	3 (9.7%)	1 (2.2%)	
*Streptococcus anginosus*	1 (1.1%)	1 (3.2%)		
*Staphylococcus epidermidis*	2 (2.1%)	2 (6%)		
Gram-negative bacilli				
*Enterobacterales*				
*Escherichia coli*	9 (9.7%)		3 (5%)	6 (40%)
*Klebsiella* spp.	10 (10.7%)	4 (13%)	6 (13%)	
*Enterobacter*, *Morganella morganii*, *Citrobacter* spp.	12 (13%)	2 (6.4%)	8 (17.4%)	2 (13.3%)
*Proteus*	1 (1.1%)			1 (6.6%)
*Serratia*	5 (5.4%)	3 (9.7%)	2 (4.4%)	
Non-fermenting GNB				
*Acinetobacter baumannii*	19 (20.4%)	5 (16.1%)	14 (25.5%)	
*Pseudomonas* spp.	6 (6.4%)	3 (9.7%)	2 (4.4%)	1 (6.6%)
*Stenotrophomonas maltophilia*	2 (2.1%)		2 (4.4%)	
Fungi				
*Candida* spp.	3 (3.2%)	3 (9.7%)		
*Aspergillus* spp.	2 (2.1%)		2 (4.4%)	

Table 3 MSSA: methicillin-susceptible *Staphylococcus aureus*; MRSA: methicillin-resistant *Staphylococcus aureus*; GNB: gram negative bacteria.

**Table 4:** Risk factors for secondary infections due to MDR pathogens.

Characteristics	MDR (52 pts)	MDS (37 pts)	*p*	OR (95% CI)	*p*
Gender (M)	38 (73.1%)	28 (75.7%)	0.976		
Age	68.2 ± 8.66	65.5 ± 9.97	0.182		
>70	24 (46.2%)	10 (27%)	0.108	2.776 (0.905-8.516)	0.074
BMI	27.76 (25.96–30.75)	26 (24.11–28.19)	0.009		
>26.7	36 (69.2%)	13 (35.1%)	0.003	4.378 (1.467–13.064)	**0.008**
Previous antibiotic treatment	34 (65.4%)	17 (45.9%)	0.107	2.450 (0.807–7.434)	0.114
Co-existing conditions					
Diabetes	14 (26.9%)	3 (8.1%)	0.051	4.802 (0.944–24.436)	0.059
Chronic/acute kidney disease	8 (15.4%)	2 (5.4%)	0.185	1.024 (0.129–8.132)	0.982
Hypertension	32 (61.5%)	15 (40.5%)	0.082	0.719 (0.208–2.489)	0.602
Chronic obstructive pulmonary disease	3 (5.8%)	3 (8.1%)	0.690		
Heart disease	12 (23.1%)	4 (10.8%)	0.228		
Malignancy	6 (11.5%)	2 (5.4%)	0.461		
Hematologic disease	0 (0%)	3 (8.1%)	0.068		
Neurological disease and mental disorder	9 (17.3%)	4 (10.8%)	0.582		
Charlson Index	3 (2.25–4)	2 (2–5)	0.053		
>2	39 (75%)	17 (45.9%)	0.010	3.451 (1.113–10.702)	**0.032**
VAP-HAP-CAP	35 (67.3%)	11 (29.7%)	0.001	1.375 (0.387–4.890)	0.623
cUTI	11 (21.2%)	4 (10.8%)	0.319		
BSI	24 (46.2%)	7 (18.9%)	0.015		

Table 4 Data are expressed as mean ± SD, median (IQR) or *n* (%). *p*-values indicate differences between deceased and surviving patients. *p* < 0.05 was considered statistically significant. Statistically significant values are expressed in bold font. In round brackets are expressed percentages and IQR, as well as the subjects analysed. HAP: hospital-acquired pneumonia. CAP: community-acquired pneumonia.

## Data Availability

Due to patient confidentiality, raw data will be available upon request with a compelling reason.
